# Pure red-cell aplasia after radioactive iodine treatment in a patient with papillary thyroid cancer and follow up for two years: a case report

**DOI:** 10.3389/fonc.2025.1539586

**Published:** 2025-06-24

**Authors:** Rui Li, Meng Wang, Shaoxin Li, Lin Liao

**Affiliations:** ^1^ Department of Endocrinology and Metabology, The First Affiliated Hospital of Shandong First Medical University and Shandong Provincial Qianfoshan Hospital, Shandong Key Laboratory of Rheumatic Disease and Translational Medicine, Shandong Institute of Nephrology, Jinan, China; ^2^ First Clinical Medical College, Shandong University of Traditional Chinese Medicine, Jinan, China; ^3^ Department of Endocrinology and Metabology, The First Affiliated Hospital of Shandong First Medical University and Shandong Provincial Qianfoshan Hospital, Shandong First Medical University, Shandong Key Laboratory of Rheumatic Disease and Translational Medicine, Shandong Institute of Nephrology, Jinan, China

**Keywords:** radioactive iodine therapy, pure red-cell aplasia, thyroid cancer, case report, papillary

## Abstract

Acquired pure red cell aplasia (PRCA) has attracted more and more attention in hematology. PRCA usually caused by infection, autoimmune diseases, thymic carcinoma, or drugs, has not been reported by radioactive iodine. Here we report a case of PRCA after radioactive iodine therapies (two times, 100 mCi and 130 mCi each) in a patient with papillary thyroid carcinoma and follow up for two years. she was treatment with cyclosporine (100mg twice per day), stanozolol (2mg three times per day) and diammonium glycyrrhizinate (150mg three times per day), returned to the normal range in 2024.

## 1 Introduction

Acquired pure red cell aplasia (PRCA) is a rare chronic severe anemia characterized by severe reduction in the number of reticulocytes in peripheral blood and the absence or near-absence of recognizable erythroid precursors in the bone marrow (1, 2). Acquired pure red blood cell aplastic anemia is most idiopathic in adults. Secondary acquired PRCA may be associated with autoimmune diseases (such as systemic lupus erythematosus), lymphoproliferative disorders (such as chronic lymphocytic leukemia), infections (such as infectious mononucleosis, viral hepatitis), thymoma and other solid tumors, or drugs (α-interferon, lamivudine etc.) or toxic substances (1, 3).

Thyroid cancer rises rapidly recent years, especially in Asia (4). Papillary carcinoma (PTC) is the most common type of thyroid cancer, accounting for about 84% (5) of all thyroid cancer. The routine therapy of PTC is surgery, thyroid hormone inhibiting therapy and radioactive iodine (RAI) therapy (internal) if necessary (6). PTC are insensitive to external radiotherapy and chemotherapy, so, RAI therapy is a very important option for PTC patients with metastasis out of thyroid.

The objective of RAI therapy is to eliminate potential remnant foci of thyroid cancer in order to minimize the risk of recurrence while improving disease-specific survival and progression-free survival (7). Although iodine 131(^131^I) is widely considered safe, it does have some side effects, such as transient neck pain and edema, gastritis, radiation thyroiditis, salivary gland dysfunction, nasolacrimal duct obstruction, and bone marrow dysfunction (8). However, PRCA has not been reported.

In this study, we reported a case of PRCA in a PTC patient after total thyroidectomy and two radioactive iodine treatments. After oral and written information, the patient still agrees to use the clinical data for research purposes and to sign an informed consent form. This study was subsequently approved by the Medical Ethics Committee of the First Affiliated Hospital of Shandong First Medical University (Qianfoshan Hospital of Shandong Province).

## 2 Case report

**Figure 1:**
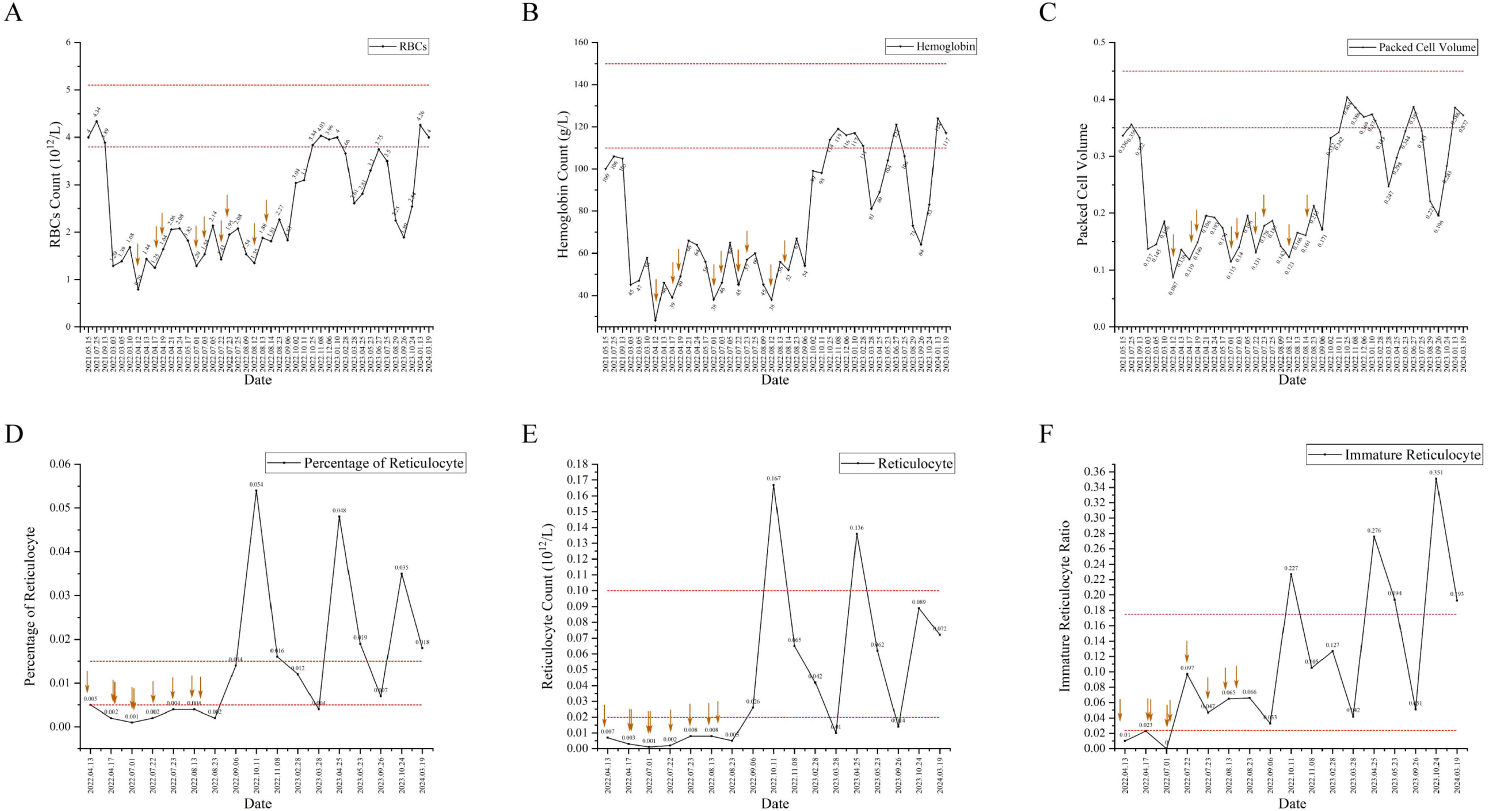
The indexes of hemocyte and reticulocyte counts analysis have changed since before thyroidectomy to now, and the interval included in the dotted line is the normal value interval. The red arrow represents blood transfusion. **(A)** The change trend of red blood cells (RBCs). **(B)** The change trend of hemoglobin (Hb). **(C)** The change trend of packet cell volume(PCV). **(D)** The change trend of percentage of reticulocyte. **(E)** The change trend of reticulocyte counts. **(F)** The change trend of Immature reticulocyte ratio.

A 51-year-old Chinese female patient with a history of PTC post-thyroidectomy was hospitalized for RAI therapy. The pathological diagnosis of the patient was papillary thyroid carcinoma, with three of the four invading the capsule. The TNM stage of PTC was T3aN1aM0. Immunohistochemical staining showed: BRAF (+). She denied the history of systemic immune diseases and thymoma, and did not take drugs that mediated red blood cell dysplasia. The series of antibodies related to rheumatic immune diseases were negative. The patient ‘s antibody series related to rheumatic immune disease was negative. Before RAI treatment, her hemocyte analysis was normal except for hemoglobin (Hb), which was almost normal(106.00g/L, female reference range: 115-150g/L), as shown in **Figure 1**. The patient’s iodine uptake rate showed that the 3h iodine uptake rate was 2.2%, the 6h iodine uptake rate was 1.9%, and the 12h iodine uptake rate was 0.8%. Because of multiple lesions, PTC volume is large, thyroglobulin 1.58ng/ml and BRAF (+), 100 mCi of 131I was administered. Two months after RAI treatment, her red blood cells (RBCs) (3.89×10^12^/L, reference range: 3.8-5.1×10^12^/L) were still normal, packet cell volume (PCV) was down to 3.32×10^-1^ (reference range: 0.35-0.45)and her Hb(105.00g/L) was almost unchanged.(**Figure 1**)

Seven months later, she was presented to hospital for further RAI therapy. Before RAI therapy, her hemocyte analysis was abnormal (RBCs 1.29×10^12^/L, Hb 45.00g/L and PCV 1.37×10^-1^). She was given prednisone acetate tablets 5mg tid, compound vitamin B tablets 1 tablet, vitamin C tablets 0.1g for temporary treatment. Thyroglobulin was 0.62ng/ml and thyroglobulin antibody was 21.20IU/ml, ultrasound of the thyroid and surrounding lymph nodes indicated thyroidectomy, the lymph nodes in the right neck region III and the left neck region IV were displayed, and the right supraclavicular lymph nodes were displayed. According to the increase of thyroglobulin antibody after the first RAI treatment (thyroglobulin antibody: 13.79IU/ml), and the indication of several cervical lymph nodes, combined with the fact that nuclear medicine doctors did not notice the patient ‘s anemia status. The next day, 130mCi of 131I was administered. The day after second RAI treatment, she was noted to have erythrocytopenia (RBCs 1.39×10^12^/L, Hb 47.00g/L and PCV 1.45×10^-1^). After RAI treatment, euthyrox was given routine treatment, plus prednisone acetate tablets 10 mg tid for three days, reduced to 5 mg tid for three days, and continued to reduce to 5 mg qd for three days before stopping treatment.

**Figure 2:**
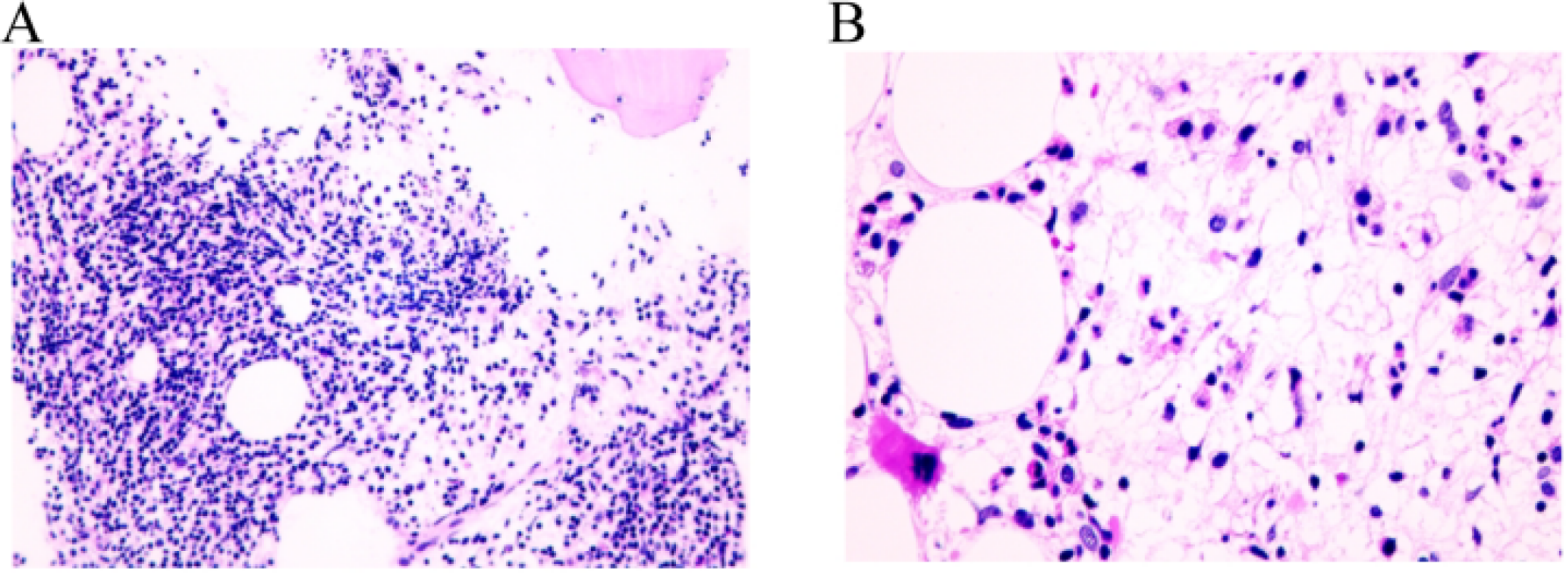
Pathological examination of bone marrow biopsy in patients with light microscope. **(A)** The proportions of granulocytes and erythrocytes increased, and granulocyte hyperplasia was dominant, MPO (more+), CD71 (less+); both granulocytes and erythroid cells were mainly mature stage cells, and there was no obvious proliferation of immature cells. CD34 and CD117 (individual+) were positive. Monocyte mild hyperplasia, lysozyme (partial+); some mature megakaryocytes were scattered, CD61(+); lymphocyte proliferation, local lymph node formation, CD20 (partial+), CD30 (more+). **(B)** No hemosiderin deposition was found; local interstitial was serous degeneration; local interstitial reticular fiber mild hyperplasia, reticular fiber staining: (local 1+~2+).

One month after second RAI treatment, she was referred to the Department of Hematology with 1 week of fatigue. Physical examination showed anemia appearance and pale eyelid conjunctiva, and about 5 cm surgical transverse incision in the neck, which healed well. No other related findings were found in physical examination. the drugs she took were levothyroxine, calcium carbonate D3 tablets, and calcitriol. Her laboratory results showed Hb drop down to 28.00 g/L, RBCs of 0.79×10^12^/L, PCV of 0.87×10^-1^. Bone marrow aspiration and biopsy was shown in **Figure 2**. A diagnosis of primary myelofibrosis was made. The patient was treated with erythropoietin injection (10000iu once every other day), meglumine adenosine cyclophosphate (180mg once per day), and transfused with red blood cells several times in view of the symptoms.

In the following 4 months, the patient was repeatedly admitted to the hospital for 4 times, and the percentage of reticulocytes and reticulocyte count were lower than the normal ranges. (**Figure 1**) The laboratory results were normal and excluded, as shown in **Table 1**. Each admission she was given erythropoietin injection (10000iu once per day), cyclic adenosine monophosphate (180mg once per day), prednisone acetate (15mg once per day), and multiple blood transfusions. She was diagnosed with PRCA on 17^th^ August 2022. The treatment was changed to erythropoietin injection (10000iu once per day), adenosine cyclophosphate (40mg once per day), prednisone acetate (10mg once per day) and cyclosporine (25mg twice per day). At follow-up, she was treatment with cyclosporine (100mg twice per day), stanozolol (2mg three times per day) and diammonium glycyrrhizinate (150mg three times per day). Her reticulocyte percentage was higher than the normal range, while Hb and RBCs count were lower than the normal range in 2023 and returned to the normal range in 2024. (**Figure 1**)

**Table 1:** Laboratory results of excluded factors.

General laboratory analyses			
Name	Value	Unit	Reference range
Ferritin	360.2	ng/mL	4.63-204
Iron	49.16	umol/L	8.95-28.64
Serum transferrin	2.04	g/L	2.02-3.36
Folate	4.17	ng/mL	3.89-26.8
Vitamin B12	287.3	pg/mL	191-946
Parvovirus B19 IgM	<0.1	index	<1.0
Parvovirus B19 IgG	19	index	<1.0
EB virus DNA determination	lower than the lower limit of detection		

Informed consent has been taken from patient and his relatives.

## 3 Discussion

Acquired pure red cell aplasia after RAI treatment is a rare phenomenon, has not been reported before. Pure red blood cell regeneration disorder is characterized by anemia, almost complete lack of red blood cell precursors in bone marrow, but usually has normal granulocyte and megakaryocyte hematopoiesis (1).

Acquired PRCA is mostly associated with autoimmune, inflammatory, infectious or neoplastic diseases, or with adverse reactions of some drugs. Approximately 50% of acquired PRCA is associated with thymoma, and less occurs in other tumors, including leukemia, malignant lymphoma, biliary adenocarcinoma, and breast cancer (3). At present, only one case of PRCA associated with thyroid cancer has been reported in 1983 (9). At the same time, in this case, we excluded autoimmune rheumatic diseases, the infection of B19 parvovirus, hepatitis B, hepatitis C, human immunodeficiency virus and EB virus according to the patient ‘s laboratory examination. We admit that we have a large dose of RAI treatment in the treatment of this patient, and do not pay attention to the hematological abnormalities before treatment and correct them in time.

Previous studies have shown that RAI treatment can lead to hematopoietic toxicity (10). In this case, the patient experienced RBCs, Hb reductions after RAI treatment. Although some studies have reported that patients have transient cytopenia after RAI treatment, PRCA has not been reported (11–14). Dereje Mengesha Berta et al.’s meta-analysis (15) showed that red blood cells and hemoglobin decreased at 3 months and 6 months after RAI treatment. The reason for the decrease may be related to the inhibition of bone marrow hematopoiesis by RIA treatment, damage to other important organs involved in erythropoiesis, and induction of oxidative stress in RBCs. These effects appear more than 2 months after treatment. Haynie et al. (11) reported transient leukopenia, anemia and thrombocytopenia after repeated administration of radioactive iodine at an interval of 3 months, and occasionally persistent anemia. Padovani et al. (12) confirmed that persistent anemia and thrombocytopenia can last for several years after RAI treatment with empirical dosing regimens. It was found that the effect of RAI on hemoglobin/hematocrit and platelet count was more significant than that on WBC count. Most patients with anemia at 1 year were also affected by other cytopenias, indicating a diffuse bone marrow effect. In addition, patients with anemia 1 year after RAI treatment also showed disease progression and multiple metastases, which may mean that anemia may also be related to bone marrow infiltration of tumors. Prinsen et al. (13) showed that the decrease of Hb in all genders was statistically significant at 3 months after RAI treatment. Hu et al. (14) showed that the decrease of Hb in all genders was statistically significant at 6 months after treatment. Duskin-Bitan et al. (10) found that WBC and Hb were inhibited when the dose of RAI was ≥150mCi. In two dosimetrically guided RAI treatment studies, high cumulative activity of RAI administered under dosimetric guidance (mean activity >250mCi) was associated with statistically significant reductions in blood cell counts (16).

Although some studies have confirmed that RAI treatment can lead to the decrease of red blood cells and hemoglobin, there is still no study to explain the relationship between RAI treatment and reticulocyte count. At the same time, there is no study to clarify the causes of severe anemia or regenerative anemia caused by RAI treatment. Based on this we speculate, RAI can cause direct bone marrow damage. Although the whole body radiation dose of ^131^I is low, a few patients may cause bone marrow microenvironment damage due to radiation sensitivity or high cumulative dose, which selectively affects erythropoiesis and causes PRCA (17). RAI may affect thyroid function, and thyroid dysfunction may indirectly affect erythropoietin secretion or erythroid differentiation, resulting in erythropoietin inhibition, resulting in PRCA (18).

The pathogenesis of acquired PRCA is related to infection, tumor and adverse drug reactions. In this case, due to our failure to correct the abnormal blood system in time and the use of a large dose of RA treatment, PRCA occurred in the patient. Therefore, according to this case, clinicians should carefully choose whether to use large doses when applying RAI treatment, and to correct hematological abnormalities in a timely manner. At the same time, patients with acquired PRCA should not only examine for the presence of thymoma, but also check for other potential tumors without thymoma, and also need to consider whether they are affected by adverse drug reactions.

## 4 Conclusions

The association between PRCA and RAI therapy for thyroid cancer is rare. Anemia usually occurs within a short period of time after RAI treatment. Blood transfusion can relieve anemia in a short time. In the treatment of acquired PRCA, prednisone acetate combined with cyclosporine is more effective. Therefore, the treatment of hormone and cyclosporine is an effective treatment method at present. Therefore, in order to avoid the recurrence of this case, clinicians should carefully choose whether to use high-dose RAI during RAI treatment, and pay attention to the patient ‘s bone marrow function and whole blood cell count, so as to detect and treat hematological abnormalities early.

## Data Availability

The raw data supporting the conclusions of this article will be made available by the authors, without undue reservation.
